# Saikosaponin D Inhibits Peritoneal Fibrosis in Rats With Renal Failure by Regulation of TGFβ1/ BMP7 / Gremlin1/ Smad Pathway

**DOI:** 10.3389/fphar.2021.628671

**Published:** 2021-10-01

**Authors:** Liu Ruiqi, Pei Ming, Su Qihang, Lei Yangyang, Chen Junli, Lin Wei, Gao Chao, Liu Xinyue, Yang Kang, Yang Hongtao

**Affiliations:** ^1^ Tianjin Academy of Traditional Chinese Medicine Affiliated Hospital, Tianjin, China; ^2^ Renal Department, First Teaching Hospital of Tianjin University of Traditional Chinese Medicine and National Clinical Research Center for Chinese Medicine Acupuncture and Moxibustion, Tianjin, China; ^3^ The First Affiliated Hospital of Henan University of Chinese Medicine, Zhengzhou, China

**Keywords:** saikosaponin d, peritoneal fibrosis (PF), TGFβ1, Bmp7, gremlin1

## Abstract

Peritoneal dialysis (PD) can improve the quality of life of patients with kidney disease and prolong survival. However, peritoneal fibrosis can often occur and lead to PD withdrawal. Therefore, it is imperative to better understand how to inhibit and slow down progression of peritoneal fibrosis. This study aimed to investigate the regulatory effect of Saikosaponin d (SSD), a monomer extracted from the plant Bupleurum, on peritoneal fibrosis and the contribution of TGFβ1/BMP7/Gremlin1 pathway cross-talk in this process. To this aim, we used a model 5/6 nephrectomy and peritoneal fibrosis in rats. Rats were divided into four groups, namely a control group (saline administration); a model group (dialysate administration; group M); a SSD group (dialysate and SSD administration); and a positive drug group (dialysate and Benazepril Hydrochloride administration; group M + A). Histological analysis indicated that peritoneal fibrosis occurred in all groups. WB, ELISA, and PCR essays suggested that TGFβ1 and Gremlin1 levels in group M were significantly higher than those in group C, whereas BMP7 expression was significantly lower. TGFβ1, Gremlin1 and BMP7 levels were significantly lower in the group where SSD was administered than in the other groups. The expression of BMP7 in SSD group was significantly increased. In addition, levels of Smad1/5/8 as assessed by PCR, and levels of p-Smad1/5/8 expression as assessed by WB were also significantly higher in the SSD group than in the M group. Expression of vimentin and α-SMA, two important markers of fibrosis, was also significantly decreased. Our study suggests a role for the TGFβ1/BMP7/Gremlin1/Smad pathway in peritoneal fibrosis with potential therapeutic implications. Finally, our results also suggest that the monomer SSD may be able to reverse peritoneal fibrosis *via* regulation of the TGFβ1/BMP7/Gremlin1/Smad pathway.

## Introduction

The incidence of chronic kidney disease has been increasing in recent years (Collaborators, 2013). As a result, the number of patients with end-stage renal disease (ESRD) is also increasing (McCullough et al., 2019) (Yamagata et al., 2015) (Hsuyuan et al., 2004). Peritoneal dialysis (PD) is a form of renal replacement therapy and an effective way to improve the quality of life and prolong survival of patients with end-stage renal disease. It is estimated that over 250 thousand patients are treated by PD worldwide. However, the complications of PD, such as peritonitis and peritoneal fibrosis, can negatively impact the therapeutic effect of PD. Peritoneal fibrosis is one of the leading causes of ultra filtration failure (UFF), which is the most common reason for PD withdrawal (Kinashi et al., 2013). Therefore, understanding how to inhibit or slow down the progression of peritoneal fibrosis is a clinical priority.

Epithelial-mesenchymal transformation (EMT) is a biological process that occurs when peritoneal mesothelial cells are damaged, resulting in a change in the normal polarity of cells. The loss of tight junctions between cells and the myofibroblast phenotype of mesothelial cells is one of the causes of peritoneal fibrosis (Stone et al., 2016) (van Baal et al., 2017). Clinical evidence for the presence of high glucose concentrations in the PD fluid is known to stimulate the EMT process. In addition, inflammation, hypoxia, and other stimuli can also increase the risk of peritoneal fibrosis (Zhou, 2019). Several clinical studies reported the occurrence of structural changes of the peritoneal membrane in patients who have received long-term continuous ambulatory peritoneal dialysis (CAPD), such as loss of mesothelial cell morphology and acquisition of a range of fibroblast characteristics due to EMT (Yáñez-Mó et al., 2003), leading to the accumulation of myofibroblasts. The expression of α-SMA is a typical feature of these cells, and its production is regulated the TGFβ pathway (Padwal and Margetts, 2016). Other pathways also play an important role in inducing EMT in the peritoneal mesothelial cells (PMC), such as activation of NFkB by angiotensin Ⅱ and activation of RhoA/Rho kinase signal by AGEs (Q. Wang et al., 2018b) (Morinelli et al., 2016). Currently, only a few treatments, such as metformin and tamoxifen, or genetic approaches such as gene knockout, have shown to inhibit EMT *in vivo* or *in vitro* in an effective way (Shin et al., 2017) (Chul et al., 2019) (Yan et al., 2018). Therefore, an urgent need remains for an effective clinical treatment of PD-associated peritoneal fibrosis.

TGFβ is a main player during fibrosis and is known to induce EMT of mesothelial cells. Bone morphogenic protein 7 (BMP7), which belongs to the BMP family of growth factors family, has also been associated with renal fibrosis (Morrissey et al., 2002) (Wang and Hirschberg, 2003). BMPs can antagonize the fibrotic effects of TGFβ by phosphorylating Smad1/5/8 and activating Smad6 (Chaverneff and Barrett, 2009) (Leeuwis et al., 2011). *In vitro* experiments have suggested that inhibition of BMP7 can stimulativee EMT of tubular epithelial cells induced by TGFβ1 (Strippoli et al., 2016).

Gremlin is a mediator downstream of TGFβ shown to promote fibrosis, and to induce the EMT process via activation of the smad pathway, thereby affecting TGFβ (Rodrigues-Diez et al., 2012) (Rodrigues-Diez et al., 2014). Prior studies suggested that Gremlin is able to promote renal, liver, and lung fibrosis (Boers et al., 2006) (Church et al., 2017) (Farkas et al., 2011). Gremlin also acts as an endogenous antagonist of BMP2/4/7 (Brazil et al., 2015) and can bind BMP [19] and prevent BMP7 from exerting its normal anti-fibrotic effect (Yang et al., 2012). A study by Zhao and colleagues has shown that the inhibitory effect of a proprietary Chinese medicine on hepatic fibrosis is associated with the suppressed expression of Gremlin and increased expression of BMP-7 (Zhao et al., 2014). However, it remains to be seen whether cross-talk between the TGFβ/BMP7/Gremlin pathways occurs during the course of peritoneal fibrosis.

Saikosaponin d (SSD) is one of the active components extracted from bupleurum, which is an herb used in traditional Chinese medicine. According to the theory of traditional Chinese medicine, the peritoneum is closely related to tri-jiao, and bupleurum can be used to treat tri-jiao disease. SSD is one of the main active triterpene saponins in bupleurum and has a steroid-like structure (Chen et al., 2016). It is mainly metabolized by the liver and although it is also metabolized by other organs. Doses of SSD that are much higher (8X) than the clinical safe dose (12.957 mg/kg) may induce acute liver injury. SSD can act on human hepatic stellate cells (Chen et al., 2016) and pancreatic stellate cells (Cui et al., 2019). It can also inhibit pro-inflammatory cytokines, improve the oxidative stress response of renal tubule cells by inhibiting ROS production, and block the cell cycle and apoptosis (Li et al., 2018). Previous studies have shown that bupleurum can protect from hepatic fibrosis and extracellular matrix deposition by inhibiting TGFβ1 and regulating IFN-g and IL-10 (Yen et al., 2005) (Liu et al., 2018). SSD has also been shown to ameliorate pancreatic fibrosis by regulating the PI3K/Akt/mTOR pathway (Cui et al., 2019). However, to the best of our knowledge, no studies in the literature have shown a therapeutic effects of SSD on peritoneal fibrosis or investigated the molecular mechanisms underlying this putative role.

In order to understand the putative effect of SSD on peritoneal fibrosis, we aimed to investigate: 1) whether a cross-talk of theTGFβ1/BMP7/Gremlin1 pathways was involved in the progression of peritoneal fibrosis; 2) whether SSD exerted inhibitory effects on peritoneal fibrosis in rat model of kidney disease; 3) whether a potential regulatory effect of SSD on peritoneal fibrosis was dependent on a TGFβ1/BMP7/Gremlin1 pathway cross-talk.

## Materials and Methods

### Animal and Cell Line Experiments

Male SD rats were purchased from Beijing Vital River Laboratory Animal Technology Co., Ltd, and kept in the Institute of Radiation Medicine, Chinese Academy of Medical Sciences, on a 12-h light/dark cycle at 22°C. All animal experiments were conducted in accordance with the US National Institutes of Health Guide for the Care and Use of Laboratory Animals and with the methods for the management of experimental animals of China. The ethical approval for this study was No. IRM-DWLL-2020100.

A total of 60 rats were used to establish the 5/6 nephrectomy model. In brief, 2/3 left nephrectomy was performed in the first week and total right nephrectomy performed in the second week, and was followed by an observation period of 4 wk. Rats were randomly divided into four groups (15 rats each) and underwent different regimens: 1) Control group (group C):15 ml of normal saline was injected intraperitoneally (ip) every day. Rats received normal saline (10 ml/kg) by gavage once daily. 2) Model group (group M): 15 ml of 4.25% dialysate was injected ip every day. Rats received normal saline (10 ml/kg) by gavage once daily. 3) SSD group: 15 ml of 4.25% dialysate was injected ip every day. Rats received SSD (5 mg/kg; with 0.5% NaCMC) by gavage once daily. 4) Positive drug group (group M + A):15 ml of 4.25% dialysate was injected ip every day. Rats were given the anti-fibrosis compound Benazepril Hydrochloride (Aoyama et al., 2002) (Yan et al., 2013) by gavage (5 mg/kg) once daily. At the end of the experiment, 10 rats were collected from each group, and test samples were randomly selected.

Human peritoneal mesothelial cells (HPMC) (HTX2481ATCC) were purchased from Otwo Biotech Shenzhen limited. Cells were cultured in a medium with fetal bovine serum (1:10), and penicillin and streptomycin (100:1). Cells were incubated at 37°C in 5% CO2. HPMCs were cultured in 96-well cell culture plates at a cell density of 3*10^4^ cells per well. Cells were divided into five groups: 1) Control group: 10%FBS; 2) Model group: 10% FBS + TGF-β1 (10 ng/ml); 3) SSD group: 10% FBS + TGF-β1 (10 ng/ml)+SSD; 4) BMP7 group: 10% FBS + TGF-β1+BMP7 (100 ng/ml); 5) Gremlin group: 10% FBS + TGF-β1+Gremlin (400 ng/ml).

### Reagents

SSD (CAS: 20874-52-6, China) was purchased from Chengdu Purifa Technology Development Co. LTD; NaCMC(C8621-25g, China) was purchased from Beijing Solebao Technology Co. LTD; TGF-β1 (AF-100-21C, United States) was purchased from PeproTech; BMP7 (Recombinant Human Bone Morphogenetic Protein 7, HY-P7008, United States) was purchased from MedChemExpress; and Gremlin (SRP4657, United States) was purchased from Sigma.

### Histological Evaluation

Peritoneal tissue was fixed in a formalin solution and embedded in paraffin. Tissue blocks sectioned, and dewaxed and hydrated prior to staining. Sections were stained with an hematoxylin solution first and then with eosin, followed by mounting in neutral resins. Masson staining was also performed. In brief, dewaxed sections were stained with Weigert hematoxylin and then with Ponceau S solution. Sections were then cleaned with phosphomolybdic acid solution and stained with aniline blue solution. Sections were inspected under a microscope (40× and 100× magnification) to assess the degree of peritoneal fibrosis.

### Immunohistochemistry (IHC) Staining

Tissue sections were dewaxed with xylene, incubated in 3% H₂O₂ for 30 min to block endogenous peroxidases, and subjected to antigen retrieval. Sections were then blocked with goat serum, incubated with a primary antibody overnight at 4°C [E-cadherin (1:50, #3195; CST), ZO-1 (1:100, ab216880; abcam)], washed in xx and subsequently incubated with a secondary antibody for 1 h at room temperature. Antibody reactions were visualized by DAB staining, followed by hematoxylin staining for 3 min. Sections were then further cleaned and sealed.

### Immunofluorescence Staining

Cells from each group were extracted and fixed, and permeabilized with Triton100. Cells were blocked with goat serum and incubated for 30 min. The primary antibody (E-cadherin and zo-1:1:100) was then added and incubated overnight at 4°C, followed by secondary antibody (1:200) incubation for 1h at room temperature. Cell nuclei were stained with DAPI. The sections were inspected under a fluorescence microscope and representative images were obtained.

### Reverse Transcription-Quantitative Polymerase Chain Reaction (RT-qPCR)

Following collection of peritoneal tissue, total RNA extraction was extracted with a TRIZOL solution. All-in-One™ First-Strand cDNA Synthesis Kit was used for reverse transcription (GeneCopoeia Cat. No.AORT-0050) according to the manufacturer’s instructions. Data quantification was carried out by 2-△△CT with ABI7500 Fast fluorescence quantitative PCR. Platinum^®^ SYBR^®^ Green qPCR SuperMix-UDG (company?) was used for amplification. Primer sequences are shown in Table1:

**TABLE 1:** Primers for reverse transcription-quantitative polymerase chain reaction analysis.

Gene	Forward Primer Sequence	Reverse Primer Sequence
Smad1	TGTTGGTGGATGGTTTCA	ACTCCTTTCCCGATGTG
Smad5	AGGACAGCCAAGCAAG	CGATCCAAAAGGAAACT
Smad8	TGCGAGTTCCCGTTTG	AGGGTAGGTGGCGTTGT
vimentin	GATGTTCGGTGGCTCC	CGGTGTTGATGGCGTC
αSAM	GGGAGTGATGGTTGGA	GGCAGGGACATTGAAG
TGFβ1	AGGAGACGGAATACAGGG	ATGAGGAGCAGGAAGGG
Gremlin1	AAGAAAGGGTCCCAAGG	TGATGATAGTGCGGCTG
BMP7	GACCCCAGAACAAGCAA	CTC​ACA​GTA​GTA​GGC​AGC​AT
GAPDH	CAAGTTCAACGGCACAG	CCA​GTA​GAC​TCC​ACG​ACA​T

### Western blot (WB)

A total of 100 mg peritoneal tissue was collected from each rat and lysed with 500 μl of cold protein lysate RIPA (Solarbio) buffer containing protease inhibitor (0.4 mM PMSF, 1 mM Iodo, 1 μM Pepstatin A). Centrifugation was performed at 14000 rpm at 4°C for 10 min, after which the supernatant was collected. Protein concentration was determined by the BCA method. Samples were loaded as 50 μg of protein in 5X loading buffer. The protein sample was separated by SDS-PAGE, transferred to a PVDF membrane (Millipore), and blocked with 5% skimmed milk solution at room temperature for 1 h. Membranes were then incubated with primary antibodies [Gremlin1 (1:1000), ab157576; Abcam; BMP7 (1:1000), ab56023; Abcam); TGFβ1 (1:1000), ab92486; Abcam; and β-Actin (1:1000), PAB36265; Bioswamp] at 4 °C overnight. On the following day, the membranes were washed and incubated with Goat anti-Rabbit secondary antibodies [(1:20000), SAB43714; Bioswamp]. After washing with TBST, the membrane was visualized using a gel imaging analyzer (FluorChem FC2 Imaging System; Alpha Innotech). Image analysis was performed using the AlphaView software (Alpha Innotech).

### ELISA

100 µl serum was added to ELISA Plate and incubated at 37°C for 1 h. The plate was then washed with xx, and incubation with 100 μl of antibody solution was performed at 37°C for 1 h. Plates were then washed three times, followed by incubation with 100 µl of ELISA antibody working solution at 37°C for 30 min. Plates were then washed again five times and incubated with 100 µl of substrate solution at 37°C for 10 min. The absorbance value was measured by a marker. The standard curve was plotted to calculate sample concentration. ELISA kits used detected BMP7 (USCNK; SEA799Ra) , gremlin1 (USCNK; SEC128Ra)and TGFβ1 (USCNK; SCA124Ra).

### Statistical Analysis

The SPSS 22.0 software was used for statistical analysis. All data were expressed as means ± standard derivation (S.D.). Data normally distributed were analyzed by one-way ANOVA, whereas data not normally distributed were analyzed by Kruskal–Wallis test. A *p*-value < 0.05 was considered as statistically significant.

## Results

### Effect of SSD on Peritoneal Fibrosis in Rats With Renal Failure

**FIGURE 1:**
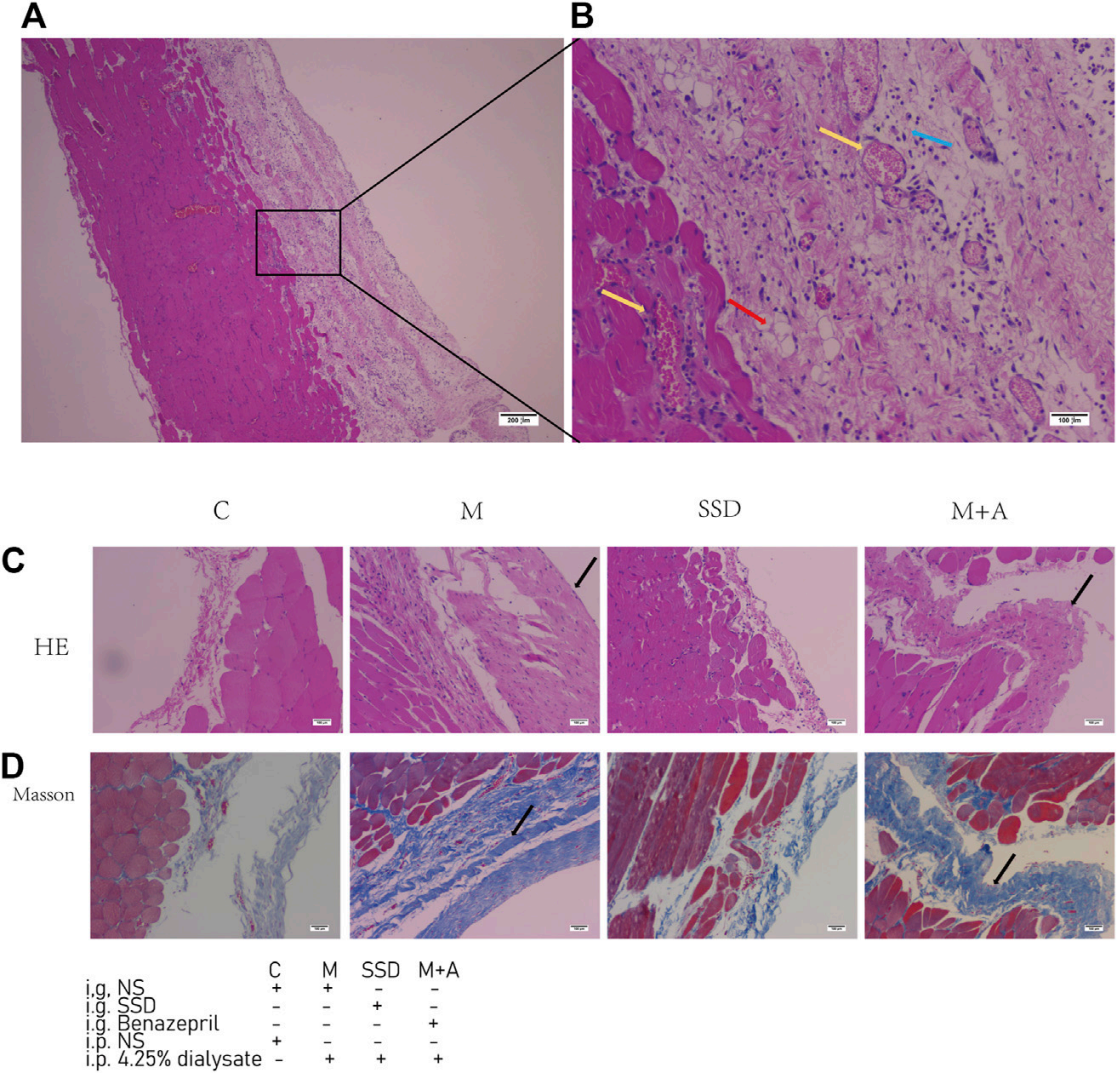
H&E staining sections and Masson staining sections of peritoneal tissue. **(A)** H&E staining of a sample in the group M **(B)** H&E staining of the same sample the group M (*n* = 6–7) at higher magnification **(C**,**D)** H&E and Masson staining of samples in the experimental groups. The yellow arrows indicate hyperplastic blood vessels. The red arrow indicates fat cells. The blue arrow indicates inflammatory cells. The black arrows indicate thickened peritoneum. Magnifications are 40× in **(A)** and 100× in **(B**–**D)**.

**FIGURE 2:**
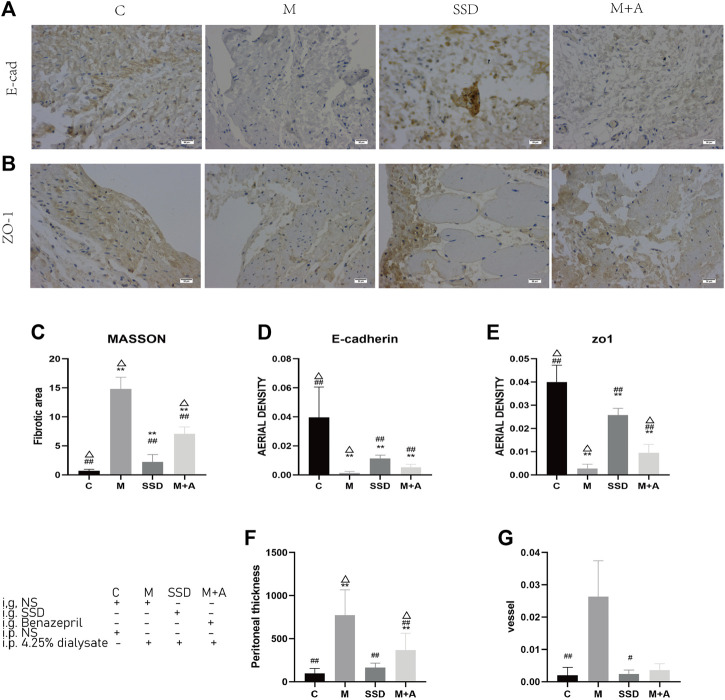
**(A**,**B)** Immunohistochemichal staining of E-cadherin (200×) and ZO-1 (200×) in the peritoneum of the experimental groups. **(C)** Semi-quantitative analysis of the average optical density of the fibrotic area, based on Masson staining **(D)** Semi-quantitative analysis of the average optical density of E-cadherin immunostaining **(E)** Semi-quantitative analysis of the average optical density of ZO-1 immunostaining. **(F)** Semi-quantitative analysis of the thickness of the peritoneum. **(G)** Semi-quantitative analysis of the richness of blood vessels (*n* = 5–7). For comparison with group M ## indicates *p* < 0.01; for comparison with the C group, ** indicates *p* < 0.01; for comparison with the SSD group△ indicates *p* < 0.01.

Peritoneal tissue sections were stained with H&E and Masson to assess the degree of histologic alterations. Peritoneal tissue samples from group C showed a smooth and thin normal peritoneum structure (Figures 1C,D). In samples from group M, in which dialysate was administered ip, the peritoneum structure was disorganized with increased thickness and obvious hyperplasia (Figures 1A,B and Figure 2F). The average thickness of the peritoneal in M group vs C group *p* = 0.000). Moreover, vascular proliferation was also present (Figures 1C,D and Figure 2G). The richness of blood vessels was significantly higher in the M group than in the C group (*p* = 0.004). In the intervention groups, both after administration of SSD (group SSD) or after administration of the anti-fibrosis compound Benazepril (group M + A), the average thickness of peritoneum was lower than that in group M, but higher than that in group C (Figures 1C,D and Figure 2F). The difference in the thickness of the peritoneum in SSD group vs M group was statistically different (*p* = 0.039). The thickness of the peritoneum in M + A group vs M group *p* = 0.000). In addition, compared with group M, the level of disorganization of the peritoneal structure (peritoneal thickening and vascular hyperplasia) was less pronounced in the SSD group than in the M + A group, (Figures 1C,D and Figure 2G). The difference in the richness of blood vessels in the SSD group vs the M group was statistically different (*p* = 0.041). Semi-quantitative analysis of Masson staining suggested that the fibrosis area in group M was not only significantly larger than that in the control group but also significantly larger than those in the other two groups (Figure 2C, all *p* < 0.01). The fibrosis area in the group SSD was also significantly lower than that in group M + A (Figure 2C, *p* = 0.000).

To further determine whether the histological alterations observed in the peritoneum were caused by fibrosis, IHC was performed to assess E-cadherin and ZO-1 expression, two factors associated with intercellular connections. Figures 2A,B shows that the brown staining area in specimens of group C is very large, and its color appears stronger than in group M. After administration of either SSD (SSD group) or Benazepril (group M + A), the brown staining area of the peritoneum was larger than that in specimens of group M, although it was smaller than that in specimens from group C (Figures 2A,B). Figures 2D,E shows semi-quantitative analysis for the several parameters measured in all groups. Based on our immunohistochemistry results, the expression of E-cadherin and ZO-1in the peritoneal tissue was significantly lower in specimens of group M compared with specimens of group C (both *p* = 0.000). In the SSD group, however, the intensity of the staining was significantly higher than that in the M group (both *p* = 0.000). These results suggest a potential role for SSD in inhibiting peritoneal fibrosis in rats.

### Role of TGFβ1/BMP7/Gremlin1 Pathway in Peritoneal Fibrosis

**FIGURE 3:**
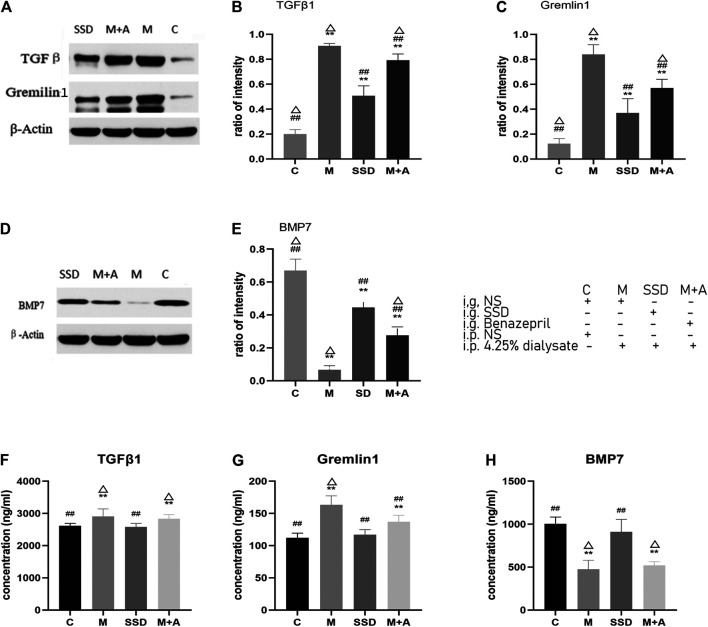
**(A)** WB of TGFβ1 and Gremlin1; β-Actin was used as the loading control. **(B,C)** Protein levels of TGFβ1 and Gremlin1 in each group. **(D)** WB of BMP7. **(E)** Quantification of protein levels of BMP7 in each group. **(F–H)** ELISA results of TGFβ1, Gremlin1, BMP7. *n* = 8 For comparison with the group M, ## indicates *p* < 0.01; for comparison with the C group, ** indicates *p* < 0.01, for comparison with the SSD group, △ indicates *p* < 0.01.

WB examined protein levels of TGFβ1, BMP7, and Gremlin1. Quantitative analysis shown in Figures 3A–C suggest that the protein levels of TGFβ1 and Gremlin1 were significantly higher in group M than in group C (both *p* = 0.000; Figures 3B,C), whereas BMP7 expression was significantly lower compared with group C (*p* = 0.000; Figures 3D,E). Following SSD administration, TGFβ1 and Gremlin1 protein levels were significantly lower than in the M group (TGFβ1 in SSD group vs M group *p* = 0.028; Gremlin1:SSD group vs M group *p* = 0.003; Figures 3B,C), whereas BMP7 level was significantly higher than that in group M (*p* = 0.001; Figures 3D,E).

To validate these results, ELISA was used to detect the level of TGFβ1, BMP7, and Gremlin1. The same trend was observed, i.e., the levels of TGFβ1 and Gremlin1 in group M were significantly higher than those in group C (TGFβ1 and Gremlin1 in C group vs M group *p* = 0.000; Figures 3F,G), whereas BMP7 levels were significantly lower than those in group C (*p* = 0.001; Figure 3H). Following SSD administration, levels of TGFβ1 and Gremlin1 were significantly lower than those in group M (TGFβ1 and Gremlin1 in SSD group vs M group *p* = 0.000; Figures 3F,G), while BMP7 expression was also significantly higher than that in group M (*p* = 0.000; Figure 3H).

**FIGURE 4:**
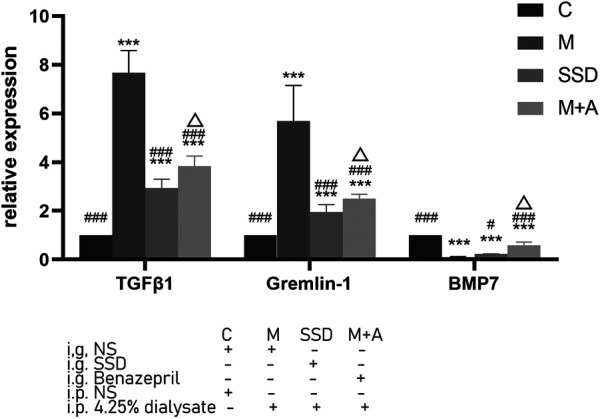
Expression levels of TGFβ1, Gremlin1 and BMP7 of peritoneal tissue by PCR. Data are presented as mean ± SD (*n* = 3). For comparison with the group C, * indicates *p* < 0.05 and *** indicates *p* < 0.001; for comparison with the group M, ### indicates *p* < 0.001; for comparison with the SSD group, △ indicates *p* < 0.001.

**FIGURE 5:**
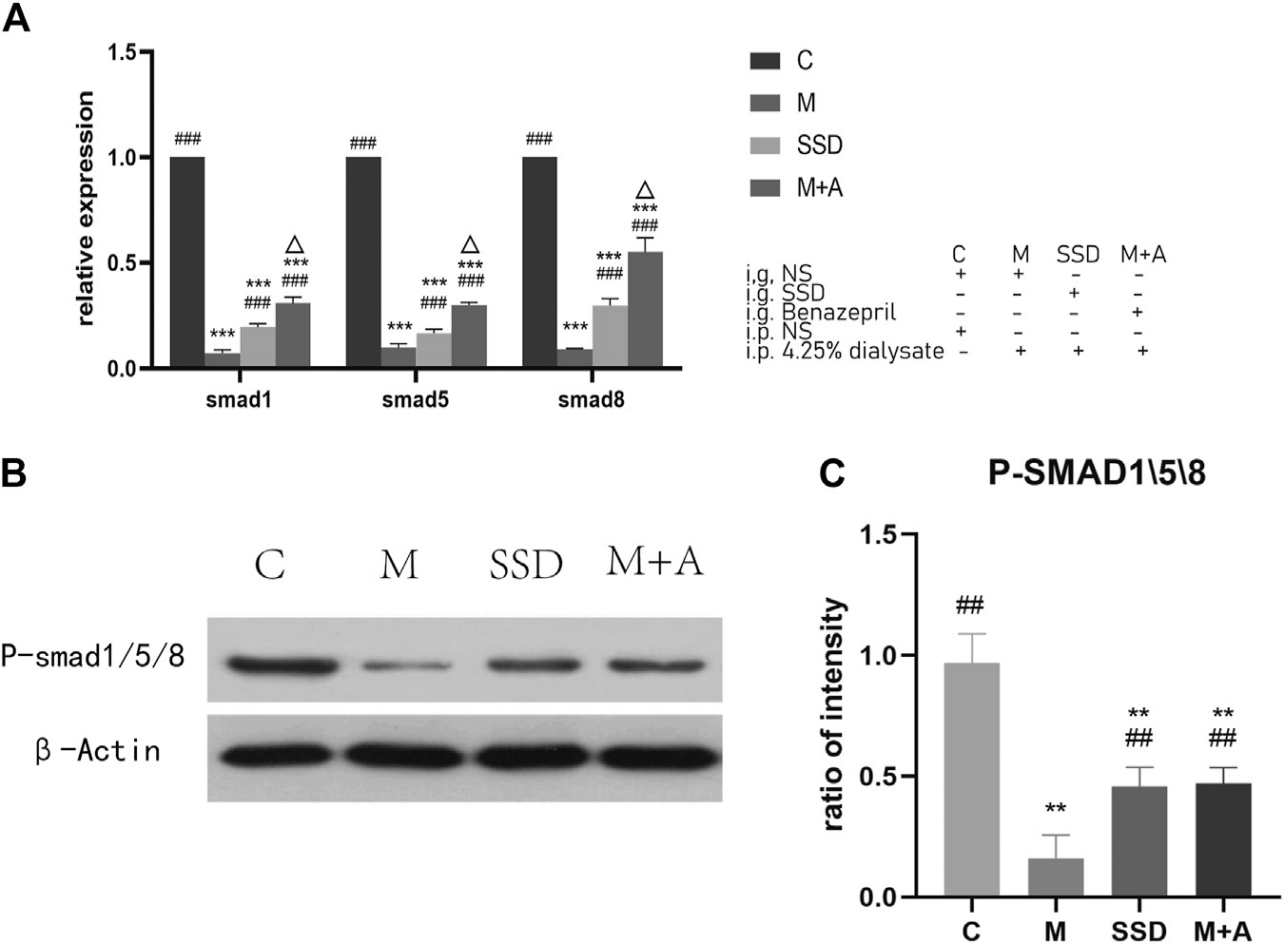
**(A)** Expression of Smad1/5/8 of peritoneal tissue by PCR. **(B)** WB of P-smad1/5/8. **(C)** Quantification of protein levels of Smad1/5/8. Data are presented as mean ± SD (*n* = 3). For comparison with the group M ## indicates *p* < 0.01; for comparison with the C group, ** indicates *p* < 0.01 and *** indicates *p* < 0.001; for comparison with the group M, ## indicates *p* < 0.01 and ### indicates *p* < 0.001; for comparison with the SSD group, △ indicates *p* < 0.01.

**FIGURE 6:**
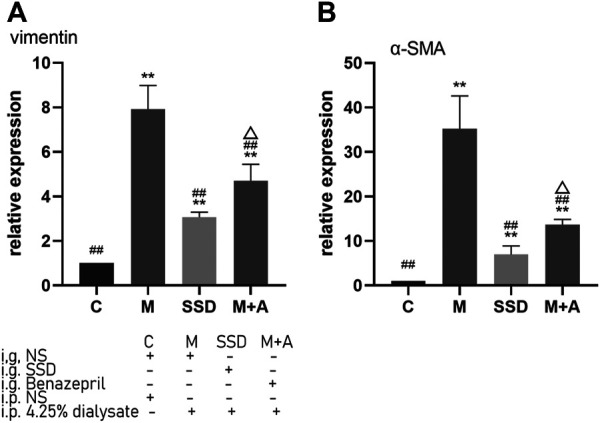
Expression levels of vimentin and α-SMA of peritoneal tissue by PCR. Data are presented as mean ± SD (*n* = 3). For comparison with the group M, ## indicates *p* < 0.01; for comparison with the C group, ** indicates *p* < 0.01; for comparison with the group M, ## indicates *p* < 0.01; for comparison with the SSD group, △ indicates *p* < 0.01.

PCR assay was also performed to detect the level of RNA expression of TGFβ1, BMP7, Gremlin1, and of the fibrosis-related cytokines Vimentin and α-SMA. Based on our PCR results, RNA levels of TGFβ1, BMP7, and Gremlin1 maintained the same trend as previously observed from WB and ELISA tests, i.e., the levels of TGFβ1 and Gremlin1 in group M were significantly higher than those in group M, while the level of BMP7 was significantly lower than that in group C (TGFβ1, Gremlin1 and BMP7 in C group vs M group *p* = 0.000; Figure 4); following administration of SSD, all these changes were significantly attenuated (TGFβ1 and Gremlin1 in SSD group vs M group *p* = 0.000; BMP7 in SSD group vs M group *p* = 0.036; Figure 4). In addition, levels of Smad1/5/8 also showed the same trend as levels of BMP7, i.e., they were significantly lower in group M than in group C (TGFβ1, Gremlin1 and BMP7 in C group vs M group *p* = 0.000; Figure 5A); whereas they were significantly higher in the SSD group than in group M (TGFβ, Gremlin1 and BMP7 in SSD group vs M group *p* = 0.000 Figure 5A). To further confirm the involvement of Smad pathway activation, p-smad1/5/8 was detected by WB. Our results showed that the expression level of p-smad1/5/8 in group M was significantly lower than that in group C (*p* = 0.000; Figures 5B,C), whereas it was significantly higher in the SSD group than in group M (*p* = 0.004; Figures 5B,C). Moreover, the RNA expression levels of vimentin and α-SMA, which are closely related to the process of fibrosis, were significantly higher in group M than in group C (both *p* = 0.000; Figure 6), but significantly lower in group SSD than in group M (both *p* = 0.000; Figure 6). These results suggest that the TGFβ1/BMP7/Gremlin1 pathway was involved in the process of peritoneal fibrosis both in the presence and absence of the SSD intervention.

### Inhibitory Effect of SSD on EMT Processes *in vitro*

**FIGURE 7:**
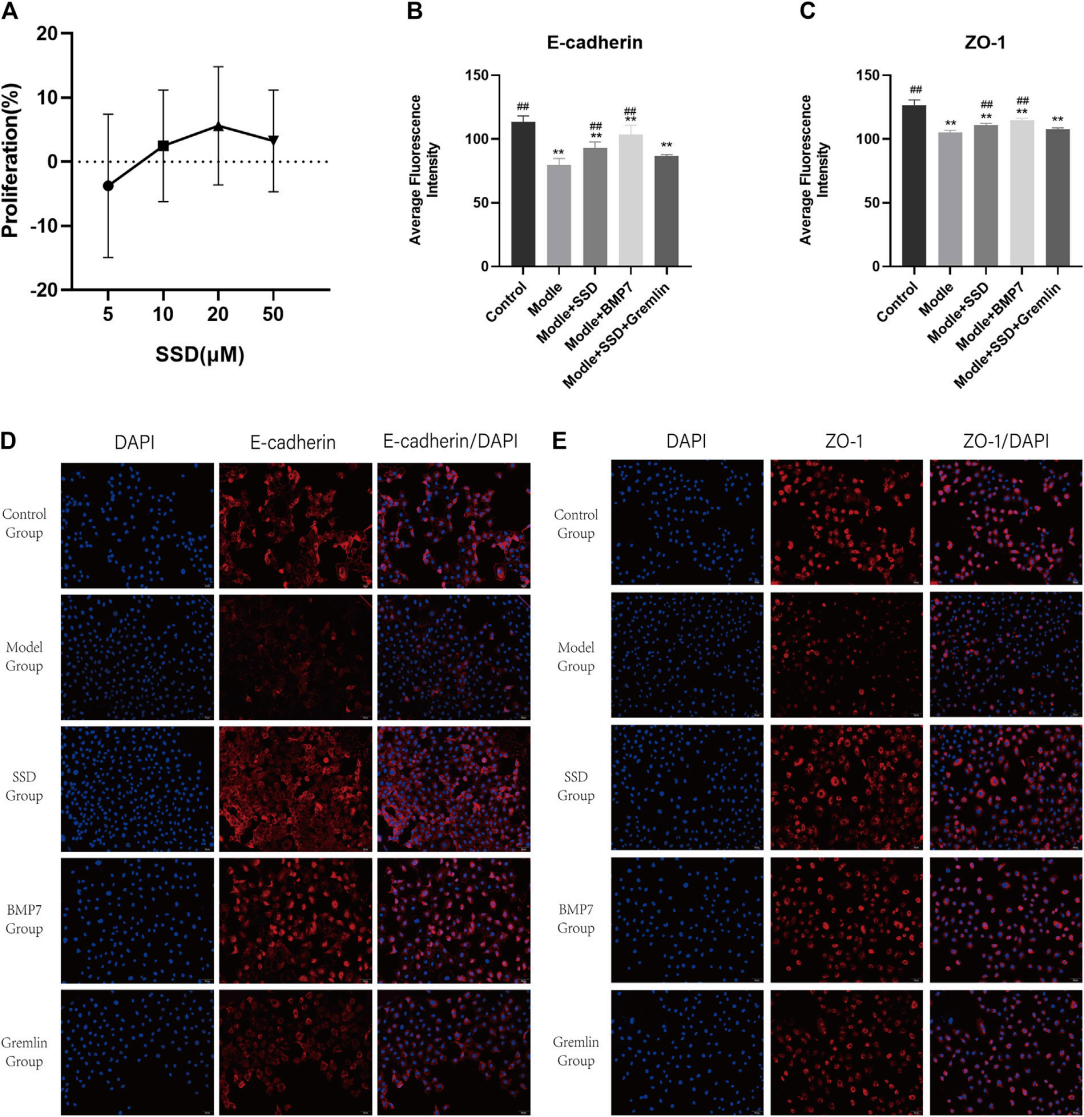
Effects of SSD on HPMC proliferation and expression levels of E-cadherin and ZO-1 detected by immunofluorescence in different HPMC groups: **(A)** MTT experiment: The proliferation rate of HPMCs interfered with TGFβ1 by different doses of SSD (5, 10, 20, 50 μM). **(B,C)** Semi-quantitative analysis of E-cadherin and ZO-1 expression levels detected by immunofluorescence. *n* = 5. For comparison with the group M, ## indicates *p* < 0.01; for comparison with the control group, ** indicates *p* < 0.01. **(D,E)** Representative immunofluorescence images (200×). Changes of EMT markers in HPMCs after interference of the TGFβ1 pathway by SSD, BMP7, and Gremlin1 are shown.

We conducted a small study to investigate the effect of SSD, BMP7 and Gremlin1 on EMT at a cellular level. Levels of E-cadherin and ZO-1, two factors associated with intercellular connections, were detected by immunofluorescence in the different groups. A preliminary MTT assay showed that cell viability was better when the SSD dose was 20 μM (Figure 7A). Compared to the control group, levels of E-cadherin and ZO-1 were significantly reduced in the model group, where interference with the TGFβ (E-cadherin in control group vs model group *p* = 0.015; ZO-1 in control group vs model group *p* = 0.000; Figures 7B,C). Expression levels of E-cadherin and ZO-1 were significantly increased in the BMP7 intervention group than in the model group (E-cadherin in the model group vs BMP7 group *p* = 0.000; ZO-1 in model group vs BMP7 group *p* = 0.001; Figures 7B,C). Administration of SSD also significantly led to TGFβ-induced reductions in levels of E-cadherin and ZO-1 (E-cadherin in SSD group vs model group *p* = 0.011; ZO-1 in SSD group vs model group *p* = 0.036; Figures 7B,C). The inhibitory effect of SSD on EMT was reversed with Gremlin1 intervention (E-cadherin in SSD group vs Gremlin1 group *p* = 0.002; ZO-1 in SSD group vs Gremlin group *p* = 0.004; Figures 7B,C). E-cadherin and ZO-1 levels in the Gremlin1 group were significantly lower than those in the control group (*p* < 0.01; Figures 7B,C).

## Discussion

SSD is a monomer extracted from Bupleurum, a plant used in traditional Chinese medicine. Bupleurum can affect “San Jiao”, which is a term related to peritoneum in traditional Chinese medicine, and is thought to be associated with the peritoneum. In addition, bupleurum is also recognized for its anti-inflammatory properties and its inhibitory effect on liver, pancreas, and renal fibrosis (Cui et al., 2019) (Liu et al., 2018) (Li et al., 2018) (Ren et al., 2020). In light of these findings, we hypothesized that bupleurum could also ameliorate peritoneal fibrosis. In this study, we have chosen one monomer in bupleurum for a more detailed investigation. Our choice of SSD, a monomer with known inhibitory effects on pancreatic fibrosis (Cui et al., 2019), is based on theories that combine traditional Chinese medicine and modern medicine concepts.

We demonstrated formation of peritoneal fibrosis following 4 wk of administration with dialysate (*via* ip injection) in a rat model of the 5/6 nephrectomy. Histological methods, in particular Masson staining, were used to evaluate fibrosis. In the model group, the structure of the peritoneum was disordered with increased thickness and obvious hyperplasia. Vascular hyperplasia was also observed (Figures 1, 2). SSD intervention resulted in an improvement of these phenotypes. In particular, Masson staining showed that the range of fibrosis was significantly lower in the SSD group than in the group M (Figures 1, 2). The average peritoneum thickness was also thinner than that of group M, while angiogenesis was barely detected in the SSD group. In a previous report, Liu and colleagues (Liu, 2004) described four crucial events leading to renal tubular EMT, i.e.,loss of epithelial adhesion properties; *de novo* expression of SMA and actin reorganization; disruption of the tubular basement membrane; and enhanced cell migration and invasion. In order to evaluate the peritoneal EMT process, we investigated the expression levels of E-cadherin and ZO1, two important markers of intercellular connections. Our results showed that E-cadherin and ZO1 levels were the lowest in group M, In the presence of SSD, however, the dialysate-induced reduction in these markers was significantly attenuated (both *p* < 0.01; Figures 1, 2). These findings are consistent with our hypothesis, suggesting that SSD inhibited the process of peritoneal fibrosis induced by dialysate in an experimental rat model of renal failure.

We then wondered whether the TGFβ1/BMP7/Gremlin1 pathway was involved in the progress of peritoneal fibrosis. Gremlin is poorly expressed in normal adult organs (Zhang and Zhang, 2009), although its expression increases when fibrosis is induced. In addition, it has been shown that Gremlin can also induce peritoneal fibrosis and blood vessel formation (Siddique et al., 2014). Indeed, we found that expression of Gremlin in the model group (both at protein and RNA level) was significantly higher than in the control group and the SSD group (Figures 3, 4). As an antagonist of BMPs, Gremlin plays different roles in a variety of cells and pathological conditions. For example, Gremlin can antagonize BMP2 in pancreatic cells and antagonize BMP7 in renal tubular epithelial cells (Marquez-Exposito et al., 2018).

Our previous studies suggested that BMP7 and Gremlin can impact organic fibrosis, such as fibrosis of heart, liver, and kidney (Yang et al., 2012) (Zhang and Zhang, 2009) (Zeisberg et al., 2007) (Protein- et al., 2007). As a TGFβ family member, BMP7 is often considered an anti-TGFβ-like factor (Phillips and Fraser, 2010) and can block fibrosis and the EMT process, in a TGFβ pathway dependent manner. TGFβ and BMP7 share similar downstream Smad signaling pathways (Meng, Chung and Lan, 2013). Previous studies have also shown that BMP7 can inhibit the expression of Smad3 and activate the expression of Smad6 (Phillips and Fraser, 2010) (Morrissey et al., 2002). In addition, substantial research focused on the BMP7/Gremlin/Smad1 pathway. However, few studies investigated the roles of Smad5 and Smad8. Moreover, while previous studies mostly focused on kidney fibrosis, liver and neurological diseases (Chaverneff and Barrett, 2009) (Leeuwis et al., 2011) (L. Wang et al., 2018a) research on a potential role of BMP7, Gremlin, and Smad1/5/8 in peritoneal fibrosis is scarce. It remains unknown whether BMP7 and Gremlin are expressed in peritoneal tissue, and whether the BMP7/Smad1/5/8 pathway in involved in peritoneal fibrosis. Therefore, we set out to investigate the role of TGFβ1/BMP7/Gremlin1 and Smad1/5/8 in the present study.

We have detected the expression levels of Gremlin1, TGFβ1, Smad 1/5/8, P-smad1/5/8 and BMP7 by several techniques including WB, ELISA and PCR. Our results indicate that, compared with the control group, expression of Gremlin1 and TGFβ1 was significantly higher in the model group, whereas the expression of Smad 1/5/8, P-smad1/5/8 and BMP7 was significantly lower (Figures 3–5). In addition, our *in vitro* results suggest that Gremlin has a potential role in reversing the action of SSD in the inhibition of the EMT process. We found that the addition of BMP7 reversed the activation of TGFβ on the cell EMT process. These findings suggest that the role of Gremlin 1 in peritoneal fibrosis may be linked to activation of the Smad-dependent TGFβ pathway. One possibility is that Gremlin1 can activate the TGFβ pathway and inhibit the expression of BMP7 and Smad1/5/8, thereby inducing peritoneal fibrosis.

The phenotypes observed in the model group were ameliorated in the SSD group. Expressions of TGFβ1 and Gremlin1, which have a fibrosis-promoting effect, were significantly reduced, both at protein and RNA level, whereas expression of anti-fibrosis markers such as BMP7, Smad1/5/8 and p-smad1/5/8 were significantly increased (Figures 3–5), suggesting that the anti-fibrotic effect of SSD is associated with BMP7 activation of Smad1/5/8. This is consistent with a previous report showing that BMP7 can activate Smad renal tubular cell (Piscione, Phan and Rosenblum, 2001). The activation of Smad1/5/8 can inhibit Smad2/3, resulting in an anti-fibrotic effect. Interestingly, the effect of SSD on Peritoneal fibrosis was significantly better than that of Benazepril (M + A group). Similarly, the effect of SSD on the expression of anti-fibrosis factors, such as BMP7 and Gremlin1, was more obvious in the group SSD than that of the M + A group (Figures 3, 4). Expression of vimentin and α-SMA, two markers of fibrotic transformation, was significantly reduced in the SSD group, further suggesting that SSD has a better anti-fibrosis effect than Benazepril (Figure 6), at least under our experimental conditions. These results were further confirmed in our *in vitro* experiments. Together, these findings suggest that SSD can effectively inhibit peritoneal fibrosis, likely in a TGFβ1/BMP7/Gremlin1/Smad pathway dependent manner.

Overall, our results suggest that the TGFβ1/BMP7/Gremlin1/Smad pathway may be a potential therapeutic target for peritoneal fibrosis. In particular, our study has also demonstrated that the monomer SSD may be able to reverse peritoneal fibrosis by regulating TGFβ1/BMP7/Gremlin1/Smad pathway. However, the correlation between the degree of peritoneal fibrosis and ultrafiltration was not detected in animal experiments performed in our study, an no correlation between treatment and ultrafiltration improvement was detected. Future experiments are necessary to validate our results and confirm the anti-fibrosis effect of SDS, as well as the molecular mechanisms underlying this potential role.

## Data Availability

The raw data supporting the conclusions of this article will be made available by the authors, without undue reservation.
